# COMPARATIVE STUDY BETWEEN ATK 1^a^ WITH CONSTRICTED POLYETHYLENE VERSUS POSTERO-STABILIZED

**DOI:** 10.1590/1413-785220253302e285178

**Published:** 2025-06-02

**Authors:** Fabiana Alves Costa Menegassi, Guilherme Blois Vasconcelos Pereira, Gabriel Alves Freitas, Rodrigo Balieiro, Sandra Tie Nishibe Minamoto, Alan de Paula Mozella, Rodrigo Sattamini Pires e Albuquerque

**Affiliations:** 1 Instituto Nacional de Traumatologia e Ortopedia, Centro de Cirurgia do Joelho, Rio de Janeiro, RJ, Brazil.; 2 Universidade Federal Fluminense, Niterói, RJ, Brazil.

**Keywords:** Arthroplasty, Replacement, Knee, Knee, Postoperative Complications, Artroplastia do Joelho, Joelho, Complicações Pós-Operatórias

## Abstract

**Objective:**

The objective of the article was to do a comparative study between Smith & Nephew ® prosthesis with constricted polyethylene against the standard in patients submitted to total knee arthroplasty surgery during a short-term follow-up. The aim was to analyze the survival of the related implants due to the range of movement and radiographic aspect.

**Methods:**

The sample was divided into two different groups: constricted polyethylene and standard polyethylene. A clinical analysis of the patients was carried out, and it was verified whether implant loosening had occurred.

**Results:**

This study evaluated 61 patients in a period of 2 years, 29 in the constricted polyethylene group. The pre-operative deformities were predominantly considered severe. In the postoperative, the tibial-femoral angle varied on average between 5 – 6° of valgus. The total range of movement in the post-operative was above 101° in both groups. One loosened implant in the constricted polyethylene group was observed.

**Conclusion:**

The patients treated with constricted polyethylene had the same range of movement as the control group. There was no significant difference between both groups related to loosened implants in short-term follow-up. ***Level of evidence III; Retrospective study.***

## RESUMO

**Objetivo:**

O objetivo da pesquisa foi realizar um estudo comparativo entre as próteses Smith & Nephew ® com polietileno constrito contra o standard, em pacientes submetidos à artroplastia total do joelho, durante um seguimento de curto prazo. Dessa forma, objetivou-se analisar a sobrevida dos implantes em questão sob o arco de movimento e o aspecto radiográfico.

**Métodos:**

A amostra foi dividida em grupos de polietileno constrito e polietileno standard. Foi realizada análise clínica dos pacientes e verificado se ocorreu soltura dos implantes.

**Resultados:**

No total, foram avaliados 61 pacientes pelo período de 2 anos, sendo 29 no grupo do polietileno constrito e 32 no polietileno standard. As deformidades pré-operatórias foram predominantemente consideradas graves. No pós-operatório o ângulo tíbio-femoral oscilou na média entre 5 e 6° de valgo. O arco de movimento total no pós-operatório foi acima de 101° nos dois grupos. Foi observada uma soltura do implante no grupo com polietileno constrito.

**Conclusão:**

Os pacientes tratados com o polietileno constrito apresentaram o mesmo arco de movimento do grupo controle. Não há diferença significativa na soltura dos implantes nos dois grupos nesse seguimento de curto prazo. ***Nível de evidência III; Estudo retrospectivo.***

## INTRODUCTION

Osteoarthritis of the knee associated with complex deformity has been a challenge for orthopedists. The integrity of the envelope of soft parts, in the case of sharp valgus and varus deviations, is usually compromised. Correction of these axial deformities is often associated with severe contractions in flexion or recurvation, requiring extensive ligament releases.^1,2^ This may result in elevation of the joint line, low patella, and possible residual instability, resulting in implant overload and premature release.^3–5^

In total knee arthroplasty surgery (TKA), instability is a major cause of prosthesis failure.^6^ According to Maynard & Sauber,^7^ acceptance of instability and implantation of non-constricted components can result in patient dissatisfaction and early review in a short-term follow-up. With its inherent stability, the poster-stabilized semi-constrict prosthesis with polyethylene constrict (MLC) has been an acceptable solution for complex cases of primary knee arthroplasties.^8^ With this, increasing the constriction of the prosthesis should reduce instability. However, it can also lead to increased transmission forces to the interface between the prosthesis and the bone, when compared to implants with the standard polyethylene, which can cause early aseptic release and a reduction in the knee arc movement.^9^

In addition to this option, the increase in constriction can also be achieved through the use of the rotary hinge prosthesis, having the advantage of being used in cases of severe bone loss, complete insufficiency of one of the collateral ligaments, ligament hyperfroxidism with excessive flex/extension spaces, neuromuscular diseases, severe rheumatoid arthritis and fixed axis deviation greater than 20 degrees.^10^ This prosthesis is an evolution of the fixed hinge models, combining flex-extension movement with rotation and thus improving movement mechanics and reducing the transmission of stress in fixation. However, in addition to presenting a greater stress transmission to the implant-bone interface, the volume of the implants requires a greater bone resection and presents a less physiological pattern of movement, with these additional disadvantages.^10^

Due to this, several authors advocate using polyethylene constricted in this type of PS prosthesis to theoretically improve the implant's stability.^8–9,11–14^

**Figure 1:**
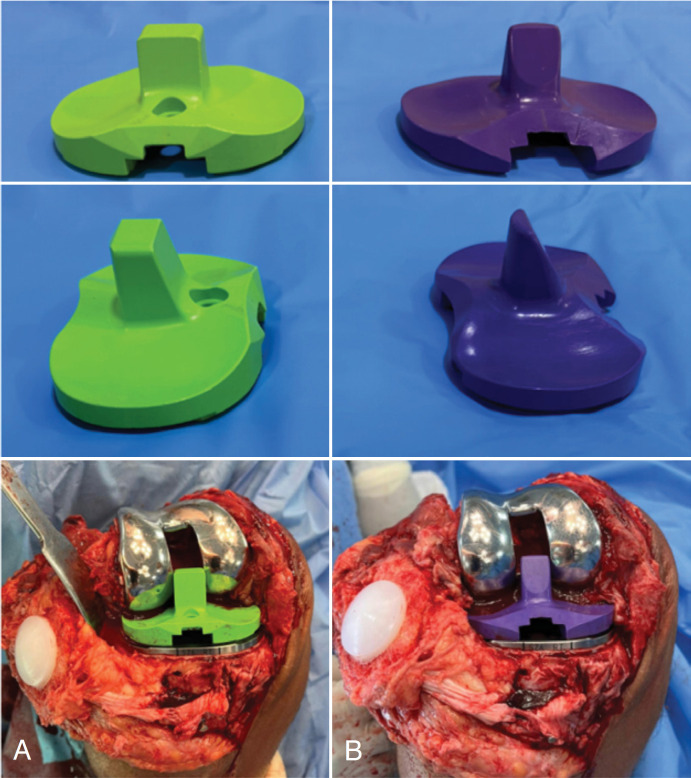
Image comparing constrained polyethylene (A) and standard (B).

The Smith & Nephew^®^ type prosthesis with constricted polyethylene is an implant that, with the increase of the pole, restricts the movements in varicose varus and valgus, and is used in some cases in our institution, which has more serious cases because it is a reference tertiary hospital in the orthopedic area (Figure 1).

The main objective of the research is to conduct a comparative study between Smith & Nephew® polyethylene-constricted prostheses and the standard in patients undergoing primary total knee arthroplasty during a short-term follow-up. Thus, the objective is to analyze the survival of the implants in question under the motion arc and radiographic aspects.

## MATERIAL AND METHODS

This study is an observational, transversal, and retrospective study. The identification of participants was carried out using data from our hospital's implant sector. Identifying the patients linked to the specific implant, it was possible to access the records of those submitted to post-stabilized primary TKA (PS) with constricted polyethylene and standard. Thus, a comparative study was conducted, observing the radiographic analysis of patients undergoing primary PS TKA, the Smith & Nephew ® brand during a follow-up of at least 2 years postoperative. The sample was divided into groups 1 with constricted polyethylene and 2 with standard polyethylene.

The sample consisted of 61 patients of both sexes and of all ages who had undergone primary TKA in the hospital with the Smith & Nephew® brand prosthesis and were admitted for treatment from 2017 to 2021. The number of patients was determined based on the observer's vision as a control case study.

The Smith & Nephew® prostheses, during this study period, were the implants offered at our hospital. Thus, the choice of the prostheses for analysis is justified, not presenting any type of conflict of interest in the evaluation.

The group was formed by 14 surgeons and doctors from the Brazilian Society of Knee Surgery, all of whom perform a minimum of 100 primary knee arthroplasties per year.

The inclusion criteria were: patients undergoing primary arthroplasty performed in the research hospital with Smith & Nephew^®^ PS type prosthesis with constricted or standard polyethylene and knee osteoarthritis. The exclusion criteria are: rheumatic diseases, failure to document medical data, use of another model of prosthesis and non-acceptance to participate in the research. The research was approved by the Institutional Ethics Council (51589621.2.0000.5273) according to the established ethical standards.

In the postoperative, with a minimum of two years of follow-up, clinical evaluations were performed by a single doctor, a Brazilian Society of Knee Surgery member. The study consisted of two experienced observers with a postgraduate (doctoral) degree in their specialty, one of whom evaluated the radiography and the other the motion arc, and no response time was stipulated to try to reproduce a more accurate evaluation.^15^

During the evaluation, the patients’ demographic data were collected, and the movement arc of the operated knee was evaluated. Angulation measurements were determined using a single goniometer with the patient naked and barefoot. The measurements were performed with the Trident^®^ brand goniometer and by the same doctor trying to reduce biases. The interval between the goniometer measurements was two degrees. The references used to measure the clinical evaluation were the center of the patella and the membrane of the femur and tibia, respectively, in frontal vision. The center of the goniometer was positioned at the center of the patella. In the sagittal analysis, the patient was diagnosed with dorsal decubitus in the examination table. The fixed arm of the goniometer was aligned with the femur diaphysis and the moving arm with the tibia diaphysis. The goniometer's joint center was positioned to coincide with the femorotibial joint center.

The radiographic analysis of the implants was performed by another orthopedist doctor with a graduate degree in radiology, who had no prior knowledge of the functional indices obtained during the initial evaluation. The X-rays were performed with bipodal support in the anteroposterior profile and axial incidences of the patella. The radiographic analysis evaluated the release of the implant using the criteria used by the *Knee Society Total Knee Arthroplasty Roentgenographic Evaluation and Scoring System.* ^16^ The evaluation of osteolysis consisted of observing the presence of a radiolucent line in the area of the prosthesement-cement or cement-bone interface, which was quantified in millimeters of thickness and subsequently analyzed in each radiographic incidence for comparison purposes. The intraoperative surgeon decided to implant with constricted polyethylene. Patients with large bone deformities or ligamental instabilities and large release of soft parts required the use of polyethylene constricted. The control group (standard polyethylene) was structured to be similar in gender and average age composition to the study group. In addition, the type of deformity of the lower limb was analyzed, and the tibio-femoral angle was measured. This angle was calculated by tracing lines between the anatomical axes of the femur and the tibia, in the pre- and postoperative period.^17^ The analysis of the radiographic data was performed using the software mDicomViewer 3.0 (Microdata, RJ-Brasil, 2007).

The medical records were analyzed, and the patients’ demographic data were collected, as well as the movement arc, body mass index (BMI), and American Society of Anesthesiology Classification (ASA). The body mass index was calculated by dividing body mass by height elevated to the square. This ratio was recorded in kilograms per square meter (kg/m^2^), as described by Adolphe Quelet.^18^

The statistical analysis was performed using Microsoft Excel 2016 and GraphPad Prism 5. Implant survival was defined as the need for revision for any cause, and release was determined by analyzing the Fischer test with a confidence interval of 95%. In addition, the Student T test of variance equality was used to calculate the outcomes analyzed with the two population-independent samples, with a significance level of 0.05.

## RESULTS

**Figure 2:**
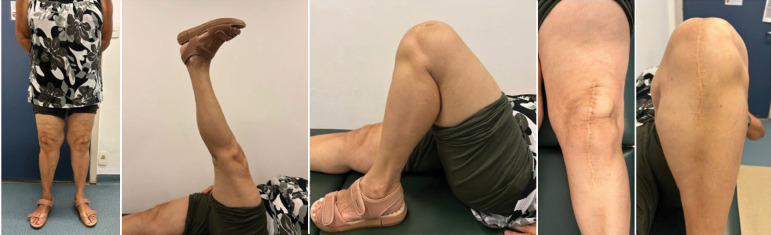
Clinical analysis with 2 years of implant evolution with constricted polyethylene.

**Figure 3:**
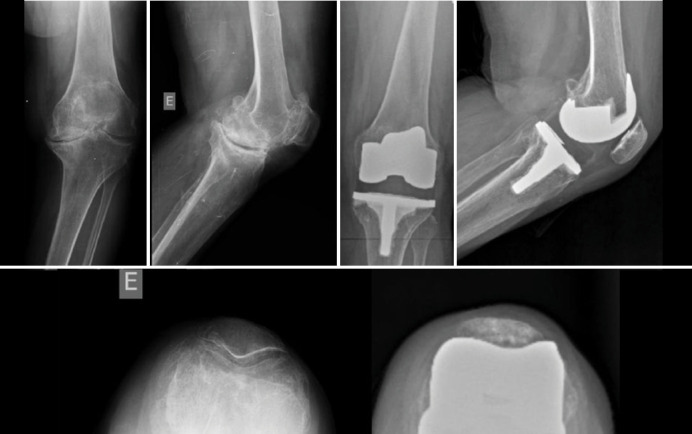
X-ray analysis with 2 years of implant evolution with constricted polyethylene.

**Figure 4:**
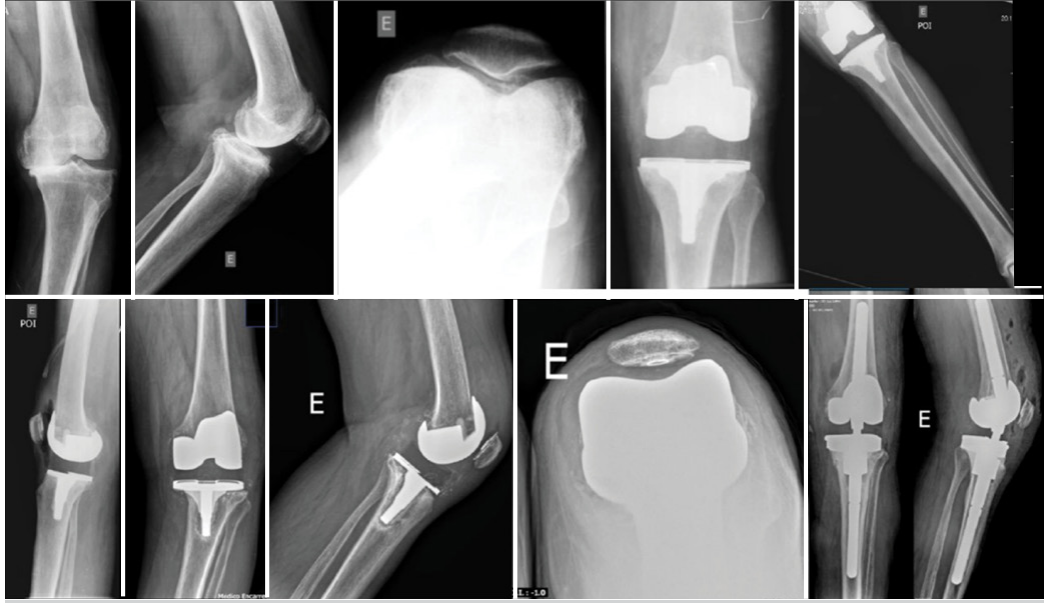
The X-ray shows the failure of the implant with constricted polyethylene, evolving with revision with a hinge-type implant.

A total of 61 patients were evaluated in postoperative primary knee total arthroplasty from 2017 to 2021. They were divided into two groups: constrained polyethylene and standard polyethylene (Table 1). All patients were diagnosed with primary knee osteoarthritis. Regarding the complications, the standard polyethylene group showed no complications. In contrast, the constricted polyethylene group presented a TKA failure with more than 2 years of follow-up, requiring a revision for a hinge-type implant (Fig. 2–4). There is no statistically significant difference between the groups (p = 0.475).

**Table 1:** Characteristics of participants.

	Constricted Polyethylene (n = 29)	Polyethylene Standard (n = 32)	P
**Sex (male)**			
Male	6 (20.7 %)	9 (28.2 %)	0.707
Female	23 (79.3 %)	23 (71.8 %)	
Age (years)	69 [64 : 73]	69 [61 : 73]	0.452
Laterality (right)	15 (51.7 %)	14 (43.7 %)	0.714
BMI	29.7 ± 5.6	32.2 ± 6.9	0.129
**ASA**			
Grade I	2 (6.9 %)	2 (6.2 %)	0.091
Grade II	23 (79.3 %)	30 (93.8 %)	
Grade III	4 (13.8 %)		
**Deformity**			
Valgus	12 (41.4 %)	9 (28.1 %)	0.413
Varus	17 (58.6 %)	23 (71.9 %)	
**Articular axis**			
Preoperative	19.4 ± 9.6	15.3 ± 8.5	0.087
Postoperative	5.7 ± 0.9	6.0 ± 1.2	0.321
**ROM**			
Preoperative	110 [80 : 112]	95 [85 : 110]	0.461
Postoperative	100 [90 : 115]	105 [90 : 117]	0.580

BMI: Body mass index, ASA: American Society of Anesthesiologists risk score, ROM: Range of movement. Categorical variables are expressed as absolute occurrence (percentage occurrence). Numerical variables with normal distribution are expressed as average ± standard deviation, and the rest as median (inter-quartile interval). The p-value refers to the comparison between groups.

## DISCUSSION

There is no study in Brazil that evaluates the polyethylene-type constricted MLC and that correlates with the movement arc of primary TKA and the survival of the implant. In addition, there are few studies in the literature on this subject. In this study, it was observed that the movement arc, the postoperative axis, the survival of implants, and the complications did not present a statistically significant difference between the groups.

It is believed that there is no waiting line for surgery in developed countries, so these patients are operated on in earlier stages of knee osteoarthritis and, consequently, are cases of less complexity. This fact makes the study extremely relevant.

When evaluating the preoperative deformities and the postoperative axis, similarity was observed between the groups, with no statistical difference.

When comparing the MLC-type implants with the PS standard prostheses, one observed a polyethylene with a higher and thicker pole, theoretically enabling a greater restriction of the implant.^12^ The prostheses with intermediate constriction MLC limit the rotation in the varus and valgus; as an advantage, they have a lower degree of constriction when compared to the knee constriction implants (CCK).^12^ The research demonstrates a positive result in the short-term follow-up with the MLC-type implant.

The decision to use the standard polyethylene or MLC was the surgeon's responsibility at the time of the operation. Other studies also conducted a similar analysis.^11,12^ It is important to emphasize that 14 surgeons and doctors from the Brazilian Society of Knee Surgery were involved in this study. With this, the intraoperative analysis under anesthesia associated with the previous radiographic examination was considered the best tool for accurately assessing each case.

The study consisted exclusively of patients with osteoarthritis of the knee. Thus, we try to uniformize the groups. Patients with rheumatoid arthritis and other rheumatic diseases were excluded from the study. Dublin and collaborators corroborated the research.^12^

This study had a short-term follow-up (> 2 years) based on other studies.^11,12^

The results show that patients in both groups have a movement arc greater than 101° in this short-term follow-up. For this reason, the MLC implant is an option in more complex cases. King et al. also observed a similar result with a one-year follow-up.^9^

All radiographic analyses were performed by a single evaluator with more than twenty years of knee surgery and a graduate degree in radiology. With this, we tried to reduce the bias of inter-observation analysis. Vivalta et al.^19^ verified that experienced observers generated individual variabilities, causing differences in the result and confusion in the literature.

In the evaluations of clinical follow-up after TKA, three parameters are considered important: the alignment of the limb, the implant positioned appropriately, and the balance of the flexion and extension spaces.^11^ This research corroborates these claims and obtains, in a general way, a postoperative alignment within normality, an implant release considered small, and an adequate movement arc.

The revision rate with the MLC implant was low, corroborating the results of other authors.^11,12^ Thiengwittayaporn et al. recommend the use of the MLC type implant when there is a varus > 19,8° or extensive release of the medial collateral ligament.^13^ This study was composed of patients with varus- or valgus deviation and a MLC type group with an average deformity of 19°.

Some studies cite the procedure of joint manipulation in patients with MLC-type implants.^11,12^ In contrast, this study did not require joint manipulation in any case. Early physiotherapeutic rehabilitation and regular postoperative control in the initial phase are believed to be fundamental for good outcomes.

The limitations of the research were: being a retrospective study, due to this, no sample calculation was made, and a small number of patients; however, the inclusion criteria were strict, and the experience of the surgeons generated a study with little index of complications. In addition, the control group comprised patients with deformities with an average of 15° in the preoperative period. Thiengwittayaporn et al. consider a severe knee deformity > 15°.^13^ Thus, this study has, in its majority, a casuistic with patients presenting severe frames in both groups.

## CONCLUSIONS

In general, the preoperative deformities were considered serious. In the postoperative period, the total amplitude of the movement arc was above 101°. The postoperative tibiofemoral angle obtained an average of between 5 and 6° valgus. There was no significant difference in the release of implants in the two groups.
